# Combination of gemcitabine, nab-paclitaxel, and S-1(GAS) as the first-line treatment for patients with locally advanced or advanced pancreatic ductal adenocarcinoma: study protocol for an open-label, single-arm phase I study

**DOI:** 10.1186/s12885-021-08275-9

**Published:** 2021-05-13

**Authors:** Chen Chang, Xiaofen Li, Dan Cao

**Affiliations:** Department of Abdominal Oncology, Cancer Center, West China Hospital, Sichuan University, No.37 Guoxue Alley, Chengdu, 610041 Sichuan China

**Keywords:** Pancreatic ductal adenocarcinoma, Gemcitabine, Nab-paclitaxel, S-1, Combination therapy, Clinical protocol

## Abstract

**Background:**

Pancreatic ductal adenocarcinoma (PDAC) is still a highly fatal malignancy among the most common cancers. More powerful treatments are expecting to bring hope for patients. Biweekly gemcitabine/nab-paclitaxel/S-1 (GAS) was proved safe and effective for patients with locally advanced pancreatic cancer in Japan. The objective of this study is to evaluate the feasibility and toxicity of GAS (repeated every 3 weeks) in the treatment of locally advanced or advanced pancreatic cancer and determine the recommended dose of S-1 in this combination.

**Methods:**

This is an open-label, single-arm, and single-center phase I trial. Patients who have been diagnosed with locally advanced or advanced PDAC pathologically without previous systemic treatments will be enrolled and be treated with GAS chemotherapy every 3 weeks (nab-paclitaxel 125 mg/m ^2^, ivgtt, day1, 8; gemcitabine 1000 mg/m^2^, day1, 8; different doses of S-1 within a dose escalation scheme) until the presence of disease progression (PD), intolerable adverse events (AEs), or requirement of patients and researchers. The primary endpoints are maximum tolerated dose (MTD) and dose-limiting toxicity (DLT). The secondary endpoints include safety, objective response rate (ORR), progression-free survival (PFS) and overall survival (OS).

**Discussion:**

This trial will adjust the administration of GAS to make it more effective for Chinese patients, while exploring the toxicity and feasibility of this adjustment.

**Trial registration:**

ChiCTR, (ChiCTR1900027833). Registered 30 November 2019.

## Background

Pancreatic ductal adenocarcinoma (PDAC) is one of the most common cancers worldwide, with a 5-year survival of less than 5% [1]. In recent years, the incidence and mortality of PDAC in China has increased year by year, surpassing the United States. Because the incidence of disease in rural areas is higher than that in urban areas, PDAC has become a major source of social disease burden in China [2]. Due to the limitations of screening techniques, more than 80% of patients diagnosed with PDAC are at the stage of unresectable advanced disease [3]. Chemotherapy is the main treatment to prolong the survival of those patients.

In 1996, gemcitabine was approved by the FDA for chemotherapy for PDAC. In 2011, study by Assaf E [4] showed that fluorouracil plus leucovorin, oxaliplatin and irinotecan (FOLFIRINOX) significantly prolonged progression-free survival and overall survival in patients with PDAC, compared with gemcitabine monotherapy. However, FOLFIRINOX had significant toxicity. A MPACT study [5] published in 2013 suggested that gemcitabine/nab-paclitaxel (AG) significantly prolonged survival in patients with advanced disease, with an objective response rate (ORR) of 23%.

The GEST study [6] in 2013 suggested that the median overall survival (OS) of S-1 combined with gemcitabine group (10.1 months) was longer than that of S-1 monotherapy group (9.7 months) or gemcitabine monotherapy group (8.8 months), and recommended S-1 as the first-line therapy for locally advanced and metastatic PDAC. In 2017, a network meta-analysis [7] suggested that S-1 showed the lowest hematologic toxicity and was superior to the previously applied FOLFIRINOX regimen.

A Phase I clinical study in Japan [8] identified the recommended dose and safety of gemcitabine/ nab-paclitaxel/S-1 (GAS, repeated every 2 weeks) as a neoadjuvant chemotherapy for patients with locally advanced PDAC, and confirmed a preliminary effect with an ORR of 31% and a disease control rate (DCR) of 94%. Considering the limited efficiency of this regimen, this study aims to evaluate the feasibility of GAS (repeated every 3 weeks) in the treatment of locally advanced or advanced PDAC, determine the recommended dose of S-1 in this combination, and explore the preliminary efficacy.

## Method

**Fig. 1:**
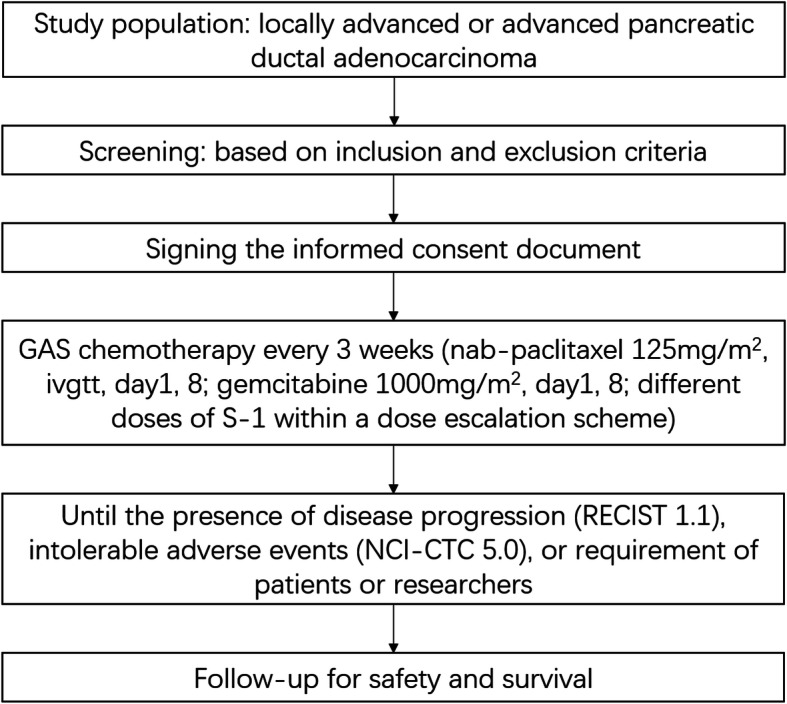
The SPIRIT flow diagram of this trial. GAS, nab-paclitaxel/gemcitabine/S-1; RECIST, Response Evaluation Criteria in Solid Tumors; NCI-CTC, National Cancer Institute Common Toxicity Criteria

The study is approved by Chinese Ethics Committee of Registering Clinical Trials (ChiECRCT20190240). The trial is registered at Chinese Clinical Trial Registry (ChiCTR1900027833). The study will be performed at West China Hospital, Sichuan University. Written informed consent will be obtained from all participants. The protocol is formulated strictly in accordance with the SPIRIT (Standard Protocol Items: Recommendations for Interventional Trials) statement [9] (Fig. 1). This protocol is the version 2.0 revised at 18 November 2019.

### Trial design

**Fig. 2:**
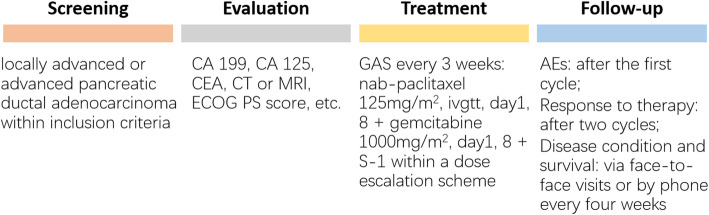
Overview of four phases of the trial. CA 199, carbohydrate antigen 199; CA 125, carbohydrate antigen 125; CEA, carcinoembryonic antigen; CT, computer tomography; MRI, magnetic resonance imaging; ECOG PS, Eastern Cooperative Oncology Group performance status; GAS, nab-paclitaxel/gemcitabine/S-1

This is an open-label, single-arm, and single-center phase I trial. Patients who have been diagnosed with locally advanced or advanced PDAC pathologically without previous systemic treatments will be enrolled. Patients will be treated with GAS chemotherapy every 3 weeks (nab-paclitaxel 125 mg/m^2^, ivgtt, day1, 8; gemcitabine 1000 mg/m^2^, day1, 8; different doses of S-1 within a dose escalation scheme) until the presence of disease progression (PD), intolerable adverse events (AEs), or requirement of patients and researchers. For patients who complete 6 cycles of the combination therapy, the subsequent therapy is determined by researchers after discussing with patients. During the trial, if the toxicity can be verified as caused by a certain drug, the dose of the drug can be adjusted. Patients who received dose adjustment more than twice should discontinue the trial. Preventive use of antiemetic drugs is allowed. Symptomatic treatments can be given when AEs develop grade 2 or greater such as fever, rash, diarrhea, and vomiting. The four phases of this trial are screening, evaluation, treatment and follow-up (Fig. 2).

### Objectives

The primary objectives:

- To evaluate toxicity and feasibility of GAS (repeated every 3 weeks)
- To establish the maximum tolerated dose (MTD) of S-1

The secondary objectives:

- To evaluate ORR using Response Evaluation Criteria in Solid Tumors (RECIST) 1.1
- To determine progression-free survival (PFS) and OS
- To analyze potential selection criteria which could be used in further studies

### Inclusion and exclusion criteria

#### Inclusion criteria

- Histologically or cytologically confirmed locally advanced or advanced PDAC;
- No previous antitumor treatment (including chemotherapy, radiotherapy, surgery or other investigational therapy);
- Aged 18 to 75 years old;
- Eastern Cooperative Oncology Group performance status (ECOG PS) ≤ 2;
- Life expectancy of greater than 3 months;
- Adequate organ function as below:
  - Absolute neutrophil count ≥1500/mm^3^, platelet count ≥80,000/mm^3^, hemoglobin ≥8.0 g/dL;
  - Total bilirubin, alanine aminotransferase, and aspartate aminotransferase ≤2.5 × upper limit of normal;
  - Serum creatinine ≤1.8 mg/dL, uric acid < 500 μmol/L, creatinine clearance ≥60 mL/min, proteinuria≤2 g/24 h;
  - Child-Pugh score<7;
  - No abnormalities in the ECG;
- Patients of both genders must be willing to practice reliable contraception during the trial;
- Must be able to participate the study voluntarily and sign the informed consent document.

#### Exclusion criteria

- Known severe hypersensitivity to any drugs of the study regimen;
- Synchronous or metachronous malignancies within 5 years (except for adequately treated basal cell carcinoma of the skin and carcinoma in situ of the cervix);
- Females who are pregnant or lactating;
- Patients with evidence of new known or suspected uncontrolled metastases to brain;
- Concurrent other kinds of chemotherapy, radiotherapy or clinical trials during the trial course;
- Serious or uncontrolled infectious disease (HIV, active tuberculosis, or HBV DNA > 103/ml), obvious coagulation disorder, active bleeding and bleeding tendency or other major diseases of the cardiovascular, respiratory, digestive, or immune system;
- Serious or uncontrolled pleural effusion or ascites;
- Severe mental disorders;
- Unsuitable for enrollment in the opinion of the investigator.

### Assessment and follow-up

The comprehensive information of eligible patients needs to be collected at least 1 week before the start of treatment, which is including: medical history, physical examination, ECOG PS score, the test of clinical chemistry, hematology and coagulation, electrolytes, the liver and kidney function, CA 199, CA 125, CEA, CT or MRI scan, and ECG. After the first cycle, AEs will be evaluated according to the National Cancer Institute Common Toxicity Criteria (NCI-CTC) 5.0. Response to therapy will be determined using RECIST 1.1 after two cycles. For patients who complete 6 cycles of the combination therapy, the subsequent therapy will be recorded. The disease condition and survival status will be recorded via face-to-face visits or by phone every four weeks.

### Sample size

As a phase I dose escalation study, we design 4 cohorts of S-1. Each dose cohort will recruit 3 patients initially. The minimum sample size will be 12 patients. Patients will be increased when dose-limiting toxicity (DLT) is found.

### Dose-escalation and limiting toxicity

#### Dose escalation scheme of S-1

The starting dose of cohort 1 (3 patients) will be 40 mg/d. MTD is determined as the highest dose that induces DLT in no more than 1 patient among a cohort of 6 patients. Patient will be recruited into 4 cohorts and will begin dosing at successively higher doses until MTD is established, or up to 100 mg/d.

The specific dose escalation scheme of S-1 is as follows:

- Cohort 1: 40 mg/d (body surface area: 1.25 ~ 1.5 m^2^)
- Cohort 2: 60 mg/d (body surface area: > 1.5 m^2^)
- Cohort 3: 80 mg/d (body surface area: > 1.5 m^2^)
- Cohort 4: 100 mg/d (body surface area: > 1.5 m^2^)

#### The determination of DLT

DLT will be evaluated after the first cycle according to the National Cancer Institute Common Toxicity Criteria (NCI-CTC) 5.0. DLT is defined as: any grade 4 hematologic toxicity; grade 3 thrombocytopenia with hemorrhage; grade 3 or greater nausea, vomiting, or diarrhea; any grade 3 or greater treatment-related nonhematologic toxicity (excluding alopecia and fatigue). (1) If there is no DLT observed in 3 patients of a cohort, the next dose cohort will be practiced; (2) If one of 3 patients experiences DLT, then we will increase 3 more patients in this dose cohort. If there is still one of 6 patients experiencing DLT, the next dose cohort will be continued. However, if more than 2 of 6 patients experience DLT, it will be reduced to the previous dose cohort; (3) If more than 2 of 3 patients experience DLT at a cohort, it will be reduced to the previous dose cohort; (4) When reducing to the previous dose cohort: if there are only 3 patients in the dose cohort, another 3 patients will be increased; if there are already 6 patients in the dose cohort, the dose will be considered as the MTD.

### Statistics

The primary objectives are to determine toxicity, tolerability, and feasibility of GAS chemotherapy for patients with locally advanced or advanced PDAC. Safety assessment includes observation and recording of any grade of AEs according to NCI-CTC 5.0. Researchers should take appropriate measures for AEs and determine the relationship between the AEs and the experimental drug. The secondary objectives are to evaluate ORR, to determine PFS and OS. In this trial, ORR is defined as complete response (CR) + partial response (PR) according to the RECIST 1.1. The 95% confidence intervals (CI) of the ORR will be calculated. PFS is defined as the time from the beginning of treatment until the first observed disease progression or cancer-related death. OS will be evaluated from the start of GAS chemotherapy to the death of patients. Kaplan-Meier method and a two-tailed log rank test are used to evaluate PFS and OS. The trial is expected to last no more than 24 months.

## Discussion

This trial aims to explore the toxicity and feasibility of the triple combination of gemcitabine + nab-paclitaxel + S-1 (repeated every 3 weeks). AG chemotherapy was confirmed that significantly improved OS, PFS, and ORR than gemcitabine in patients with metastatic PDAC in both phase 1–2 and phase 3 trials [5, 10]. Thus, AG was recommended by National Comprehensive Cancer Network (NCCN) as the first-line chemotherapy regimen for metastatic disease [11]. Nab-paclitaxel showed synergistic antitumor activity when combined with gemcitabine and improved the concentration of gemcitabine in tumor [12]. The most common adverse events reported during AG therapy were peripheral neuropathy and myelosuppression. S-1 is an oral fluoropyrimidine derivative, widely applied in gastric, colorectal, and other cancers, which enhance the anticancer activity of 5-FU and meanwhile reduce its toxicity [13]. In GEST study [6] S-1 was recommended as the first-line therapy for locally advanced and metastatic disease. Based on these promising findings, GAS as a neoadjuvant chemotherapy for patients with locally advanced PDAC was firstly tested in Japan [8], and confirmed a preliminary effect with an ORR of 31%. This trial will adjust the application cycle of GAS to make it more suitable for Chinese patients, while exploring the toxicity and feasibility of this adjustment. GAS chemotherapy is expected to increase the chance of surgical resection for patients with locally advanced and advanced disease to prolong survival.

## Data Availability

The datasets generated and analyzed during the trial are available from the corresponding author within reasonable request.
